# Cell Stiffness Is a Biomarker of the Metastatic Potential of Ovarian Cancer Cells

**DOI:** 10.1371/journal.pone.0046609

**Published:** 2012-10-04

**Authors:** Wenwei Xu, Roman Mezencev, Byungkyu Kim, Lijuan Wang, John McDonald, Todd Sulchek

**Affiliations:** 1 The George W. Woodruff School of Mechanical Engineering, Georgia Institute of Technology, Atlanta, Georgia, United States of America; 2 School of Biology, Georgia Institute of Technology, Atlanta, Georgia, United States of America; 3 The Wallace H. Coulter Department of Biomedical Engineering, Georgia Institute of Technology, Atlanta, Georgia, United States of America; University of Nebraska Medical Center, United States of America

## Abstract

The metastatic potential of cells is an important parameter in the design of optimal strategies for the personalized treatment of cancer. Using atomic force microscopy (AFM), we show, consistent with previous studies conducted in other types of epithelial cancer, that ovarian cancer cells are generally softer and display lower intrinsic variability in cell stiffness than non-malignant ovarian epithelial cells. A detailed examination of highly invasive ovarian cancer cells (HEY A8) relative to their less invasive parental cells (HEY), demonstrates that deformability is also an accurate biomarker of metastatic potential. Comparative gene expression analyses indicate that the reduced stiffness of highly metastatic HEY A8 cells is associated with actin cytoskeleton remodeling and microscopic examination of actin fiber structure in these cell lines is consistent with this prediction. Our results indicate that cell stiffness may be a useful biomarker to evaluate the relative metastatic potential of ovarian and perhaps other types of cancer cells.

## Introduction

The mechanical integrity of cells is regulated by a dynamic network of structural, cross-linking, and signaling molecules [1]. Therefore, alterations of mechanical properties of individual cells can reveal important information about changes in these networks. Studies of a variety of diseases utilizing different experimental techniques have shown that abnormalities in the elastic properties of cells are associated with disease pathogenesis and progression [2], [3], [4], [5], [6], [7], [8], [9], [10], [11], [12], [13], [14], [15], [16], [17]. For example, invasive tumor cells mechanically soften and modify their adhesion to extracellular matrix, which enhances their capacity to escape the primary tumor [5], [17], [18]. Measurements of cancer cell stiffness, quantified by the Young’s modulus, have shown a strong correlation between cell deformability and cell malignancy [5]. Similarly, the stiffness of metastatic cancer cells isolated from the pleural fluids of breast cancer patients was reported to be more than 70% lower, with a standard deviation over five times narrower, than benign reactive mesothelial cells [3].

The distribution of the actin network plays an important role in determining the mechanical properties of single cells [19], [20], [21]. As cells transform from non-malignant to cancerous states, their cytoskeletal structure changes from an organized to an irregular network, and this change subsequently reduces the stiffness of single cells [5], [22]. The studies of mechanical properties of cancer cells discussed above imply that change of stiffness of single cells can indicate the presence of malignancy [15], [16], [23], [24].

The need for effective biomarkers for diseases is particularly important in the case of ovarian cancer, which is the most lethal of gynecological cancers. Ovarian cancer was ranked fifth among leading causes of cancer-related deaths of U.S. women in 2007 and its 5 year survival rate was 46% for all cases diagnosed within 1999–2005 [25]. Due to the unavailability of reliable screening in clinical practice and the asymptomatic course through early stages of the disease, the majority of ovarian cancer cases (68%) are diagnosed as metastatic disease with poor survival [26].

In this study of the mechanical properties of cells from several different ovarian cancer cell lines and non-malignant immortalized ovarian surface epithelial cells (IOSE), we demonstrate that cell stiffness not only distinguishes ovarian cancer cells from non-malignant cells, but also can distinguish more tumorigenic/invasive cancer cells from less tumorigenic/invasive types. Our findings indicate that measurement of cell stiffness of ovarian and perhaps other types of cancer cells may not only contribute to a better understanding of the physical and molecular mechanisms underlying tumor progression, but may also serve as a useful clinical tool in the assessment of metastatic potential.

## Materials and Methods

### Ovarian Cell Line Growth and Sample Preparation

Immortalized ovarian surface epithelial cells (IOSE) were generously provided by Dr. N. Auersperg (University of British Columbia, Vancouver, Canada) and cultured in 199/105 medium (1∶1) supplemented with 15% fetal bovine serum (FBS, Atlanta Biologicals, Atalanta, GA) and 1% antibiotic-antimycotic solution (Mediatech-Cellgro, Manassas, VA). The ovarian cancer HEY and HEY A8 cell lines were provided by Dr. G. Mills (MD Anderson Cancer Center, Houston, TX) and grown in RPMI-1640 supplemented with 10% FBS and 1% antibiotic-antimycotic solution (R10 medium). The ovarian cancer OVCAR-3 and OVCAR-4 cell lines were procured from the Developmental Therapeutic Program (DTP) of the National Cancer Institute (NCI) (Bethesda, MD). Before AFM experiments, cells were plated into a Fluorodish (World Precision Instruments, Sarasota, FL) with an initial density of 10,000–20,000 cells/cm^2^.

### Atomic Force Microscopy

We conducted atomic force microscopy (AFM) mechanical measurements [27], [28] on single ovarian epithelial cells. The AFM used in our experiments is the MFP-3D (from Asylum Research, Santa Barbara, CA) with a combined Nikon Ti inverted optical microscope (Nikon, Melville, NY) used to optically align the probe to the cells. The probes used in this study were MCST-AUHW (Bruker, Camarillo, CA) with a nominal spring constant of 0.03 *N*/*m*. To simplify the contact geometry and minimize the lateral strain of the sample during indentation, the cantilever tip is modified by attaching a plain silica microsphere of diameter 4.7 µm. Measurements were conducted in cell culture media at room temperature, with cells plated on the glass bottom of the Fluorodish. To eliminate the confounding effects of neighboring cells on cytoskeleton arrangement and morphology, single cells were measured.

**Figure 1:**
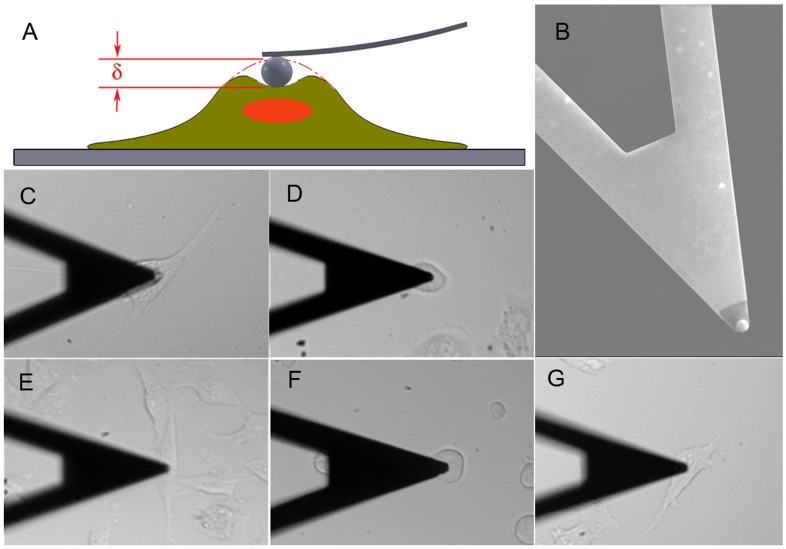
Schematics of experiments. (A) Sketch of measurements on cells with AFM, *δ* is indentation, (B) SEM image of the beaded tip, stiffness measurements of single cells with AFM for (C) IOSE, (D) OVCAR-4, (E) HEY, (F) OVCAR-3 and (G) HEY A8 cells. Same cantilever was used; the arm of the cantilever has a width of 20 µm and serves as a scale bar.

Prior to cell measurements, the cantilever was calibrated on the glass bottom of the Fluorodish using the thermal vibration method [29] with the resultant thermal spectrum fitted with Lorentzian function to determine the spring constant. The cells were indented approximately over the perinuclear region of individual cells. The indentation depth was chosen to be at least 1 µm in order to better simulate deformations which occur physiologically. The force versus indentation curves from each measurement were analyzed using a Hertzian contact model [30], [31] to obtain the Young’s modulus of each cell. A sketch of the experimental set up is shown in Fig. 1 *a*. Scanning electron micrograph of the beaded tip used in this experiment is shown in Fig. 1 *b*. Examples of optical images obtained during cell indentation are shown in Figs. 1, *c*-*g*.

### Fluorescence Imaging and Analysis of F-actin

We imaged the labeled F-actin network of each cell line using fluorescence microscopy. Cells were grown on a glass coverslip to a density of 5,000 cells/cm^2^. The cover slip with plated cells was placed in a well of a 6-well plate with 2 mL cell culture media. The cells were incubated at 37°C overnight and then stained with fluorochrome-conjugated phalloidin. The cells were stained by first fixing with 1 mL 4% formaldehyde in PBS (pH 7.4) for 10 min and permeabilizing with 1 mL 0.2% TX-100, blocking with 1% BSA for 20 min and incubating for one hour with 1∶20 Alexa Fluor 546 phalloidin (Life Technologies, Grand Island, NY) in 1% BSA. All steps during the staining process were conducted at room temperature in a dark room. After staining, the cells were sandwiched between the glass coverslip and a glass slide, mounted with ProLong Gold and sealed with nail polish. Multiple images of each cell line were taken using a Nikon Ti microscope (Nikon, Melville, NY) with the TRITC excitation/emission filter set. The analysis was limited to single cells which were not in contact with other cells.

The structural characteristics of the actin network were analyzed by quantifying an orientation parameter for actin filaments. A two-dimensional Fast Fourier Transform (FFT) was applied to the original fluorescence images using MATLAB routines (The MathWorks, Natick, MA). From the transformed image, custom MATLAB codes calculated the angular amplitude of the FFT by summing the square of the FFT components, from which the orientation distribution of actin filaments was determined as a function of angle. The mathematical algorithms calculating the orientation distribution function were based upon those reported previously in the literature [32]. The method is illustrated in Fig. S1 in the supporting material with a representative fluorescence image of an IOSE cell.

### Migration and Invasion Assays

The CytoSelect 24-well cell migration and invasion assay kit (Cell Biolabs, San Diego, CA) was used according to the manufacturer's instructions. For the migration assay, 1.5×10^5^ cells in serum-free DMEM/F12 medium containing 0.5% BSA, 2 mM CaCl_2_ and 2 mM MgCl_2_ were loaded into individual uncoated inserts with approximately 8 µm pore size. The inserts were placed in a 24-well plate containing RPMI-1640 medium supplemented with 10% FBS. After 3 h incubation at 37°C in humidified air with 5% CO_2_, the cells that migrated to the underside of the inserts were detached, lysed and quantified using CyQuant GR fluorescent dye on a plate reader at 480 nm/520 nm (Synergy 4, BioTek, Winooski, VT). Invasion assays were carried out in an identical manner with 32 h incubation using basement membrane matrix-coated inserts. All assays were carried out in triplicates with an initial time course study conducted to reach significant transmigration.

### Microarray and Pathway Enrichment Analysis

RNA was extracted from two non-confluent cultures of HEY and HEY A8 cells grown in R10 medium using Arcturus PicoPure RNA Isolation Kit (Applied Biosciences, Carlsbad, CA) according to the manufacturer’s instructions and RNA integrity was verified using a Bioanalyzer RNA Pico Chip (Agilent Technologies, Santa Clara, CA). mRNA was labeled using the IVT Labeling Kit (Affymetrix, Santa Clara, CA) and biotin-labeled mRNA was hybridized on GeneChip Probe Arrays U133 Plus 2.0 (Affymetrix). Affymetrix.CEL files were processed using the Affymetrix Expression Console Software version 5.0 using the RMA 3′-expression workflow. The 4,746 features with lowest 10% values of the logarithm of signal intensities across all 4 chips were removed and the remaining 49,929 features were analyzed using Significance Analysis of Microarrays (SAM) version 4.0 [33] with following parameters: Response type: two-class unpaired; Test statistic: T-statistic; Number of permutations: 500; Data in log2 scale; No median centering. Genes were reported as differentially expressed between HEY and HEY A8 classes if they met following criteria: (i) False Discovery Rate = 1.1% and (ii) absolute fold change (FC) ≥1.5.

Biological interpretations of the differential gene expression data were performed by pathway enrichment analysis using MetaCore 5.2 (GeneGO, St Joseph, MI). Significantly perturbed pathways and networks were identified by mapping up-regulated and down-regulated genes (combined and individually) onto GeneGO canonical pathway maps (collection of manually curated signaling and metabolic pathways) and GeneGO process networks (manually curated network models of main cellular processes) [34].

GeneGO canonical pathway maps and process networks were ranked according to their relevance to the input set of genes using p-values calculated based on a hypergeometric distribution. Multiple testing correction was performed using False Discovery Rate with the adaptive threshold set to permit no more than 1 pathway/network incorrectly predicted as significantly enriched.

To further extend biological interpretation of the differential gene expression data, the topological significance analysis (TSA) of gene expression profile was performed using online tool provided by GeneGO Inc (http://topology.genego.com/zcgi/topology_scoring.cgi). This tool maps differentially expressed genes onto a GeneGO proprietary database of protein-protein interactions and identifies proteins that occupy topologically significant positions with respect to differentially expressed genes [35], [36]. Topologically significant genes (p<0.01) were identified for all genes up-regulated in HEY A8 cells relative to HEY cells using the “transcriptional activation paths from all nodes” algorithm and subsequently mapped to GeneGO canonical pathway maps as described above.

### qPCR

Selected genes (PRKAA2, TWF1 and MYLK), identified by microarray analysis as significantly differentially expressed between HEY A8 and HEY cells, were validated using predesigned TaqMan® Gene Expression Assays (Life Technologies, Grand Island, NY). RNA was extracted from 3 non-confluent cultures of HEY and HEY A8 cells (Arcturus PicoPure RNA Isolation Kit) and reverse-transcribed and amplified using Applause 3′-Amp system (NuGen Technologies, Inc., San Carlos, CA) according to the manufacturer’s instructions. qPCR assays were performed for each gene and each sample in 4 replicates using thermal cycling conditions recommended for TaqMan® Gene Expression Master Mix and fold change values between Hey A8 and Hey cells were determined using 2^−ΔΔCt^ method using GAPDH gene as internal control.

### Statistical Analysis

Overall statistical significance of differences in mean stiffness among cell types was tested using the Kruskal-Wallis test. Significance of differences between all pairs of cells was tested using Dunn’s post test. Significance of differences in migration and invasion among IOSE, HEY and HEYA8 cells were tested by ANOVA, followed by Tukey’s test for pairwise comparisons (*p<0.05; **p<0.01; ***p<0.001. Kruskal-Wallis test, ANOVA and all post tests were performed using GraphPad Prism version 5.02 for Windows (GraphPad Software, San Diego, CA). Associations between cell stiffness and cell invasiveness, cell stiffness and cell migratory properties, and cell stiffness and the degree of co-alignment of F-actin were tested using Pearson’s product-moment correlation and Spearman’s rank correlation and expressed as correlation coefficients (r and ρ, respectively). Correlation analysis was performed using the free statistical software R [37].

## Results and Discussion

### Cell Stiffness is a Biomarker of Metastatic (Migratory/Invasiveness) Potential

**Figure 2:**
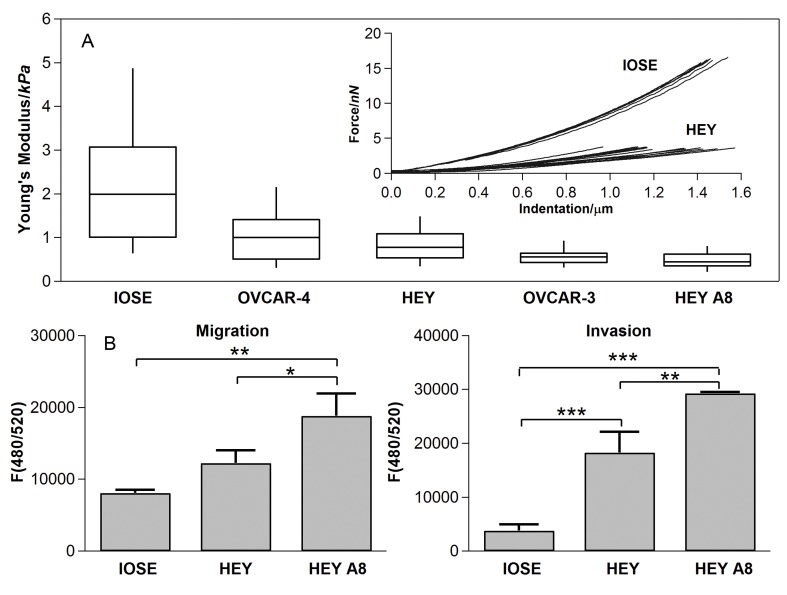
Stiffness distribution of cells and results of migration and invasion test. (A) Box-and-whisker plots of stiffness of single cells for different cell lines, the percentiles are 10%, 25%, 50%, 75% and 90%, the inset shows the representative force curves of IOSE and HEY. Overall difference among means is significant (p-value<2.2×10^−16^, Kruskal-Wallis); pairwise differences are significant between IOSE and HEY, HEY A8 and OVCAR-3 cells, between HEY A8 and HEY cells and between HEY A8 and OVCAR-4 cells (p<0.05, Dunn’s post test); (B) Migration and invasion tests for IOSE, HEY and HEY A8 cells. F(480/520) is a fluorescence intensity at 480 nm excitation and 520 nm emission, which is proportional to the number of migrating or invading cells.

Representative force-indentation curves obtained from mechanical probing of individual cells are plotted in Fig. 2 *a*. Each curve represents the applied force necessary to indent an individual ovarian cell from the IOSE and HEY cell lines. The probe contacts the cell at an indentation of 0 µm. Since the slope at a point of each force-indentation curve is related to the cell stiffness, variability of slopes for each probed cell in a given cell line indicates variability of stiffness among individual cells from the same culture. In general, curves corresponding to non-malignant IOSE cells have larger slopes than those corresponding to ovarian cancer HEY cells and are therefore stiffer.

The force curves were analyzed with a Hertzian contact model to determine the corresponding Young’s modulus of individual cells. We determined the Young’s modulus for different ovarian epithelial cell lines, including non-malignant IOSE and a variety of cancer cell lines (OVCAR-3, OVCAR-4, HEY, and HEY A8). The distribution of Young’s modulus of individual cells from different cell lines is depicted in the box and whisker plots (Fig. 2 *a*). IOSE demonstrated higher mean stiffness than any of the ovarian cancers. The overall difference among cell lines was significant (p<2.2×10^−16^) with the following pairs displaying significant differences (p<0.05): IOSE vs OVCAR-3, IOSE vs HEY, IOSE vs HEY A8, OVCAR-4 vs HEY A8, and HEY vs HEY A8. The mean Young’s moduli and standard deviations of these cells are summarized in Table 1. The non-malignant IOSE cells demonstrated higher intrinsic variability in cell stiffness than cells from any ovarian cancer cell line, which is consistent with the previously reported higher variability in stiffness of benign cells relative to breast cancer cells isolated from pleural fluids [3].

**Table 1:** Mean Young’s moduli and corresponding standard deviations, with sample size in parentheses.

	IOSE(55)	OVCAR4(18)	HEY(60)	OVCAR3(20)	HEYA8(59)
**Mean stiffness/kPa**	2.472	1.120	0.884	0.576	0.494
**Standard deviation/kPa**	2.048	0.865	0.529	0.236	0.222

Dunn's Multiple Comparison Test	Difference in rank sum	Significant? P<0.05?	Summary
IOSE vs OVCAR-4	46.17	No	ns
IOSE vs HEY	55.91	Yes	***
IOSE vs HEYA8	105.7	Yes	***
IOSE vs OVCAR-3	91.40	Yes	***
OVCAR-4 vs HEY	9.742	No	ns
OVCAR-4 vs HEYA8	59.49	Yes	**
OVCAR-4 vs OVCAR-3	45.23	No	ns
HEY vs HEYA8	49.75	Yes	***
HEY vs OVCAR-3	35.49	No	ns
HEYA8 vs OVCAR-3	−14.26	No	ns

Tests of significance tests between different cell populations are also included.

ns: not significant; **: p<0.01; ***: p<0.001.

**Figure 3:**
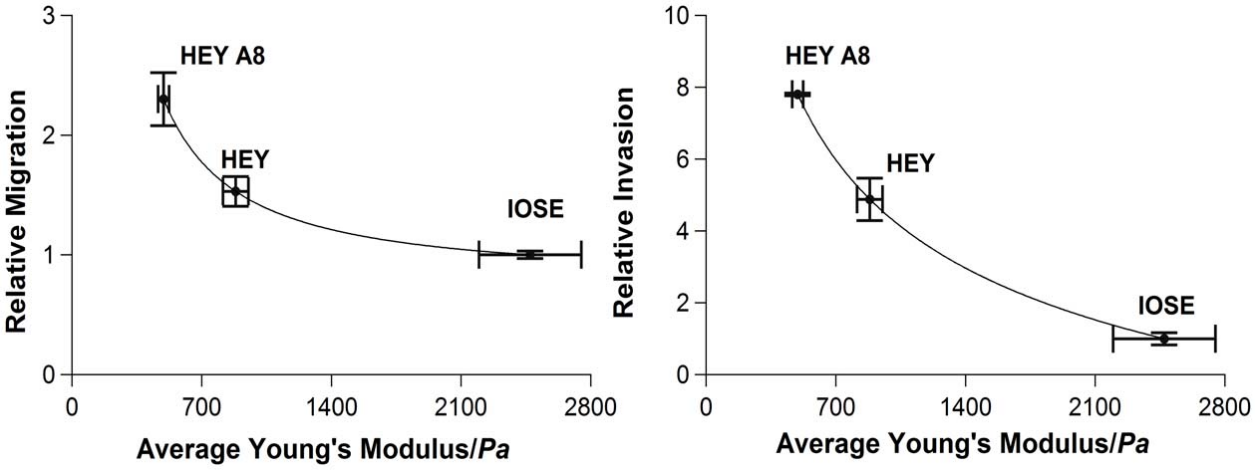
Scatterplots of relative migration and invasion versus average stiffness for IOSE, HEY and HEY A8 cells (migration and invasion of IOSE cells = 1). The data points are fitted with power law for clarity. Error bars: standard errors of means.

Notably, HEY and HEY A8 cells derived from the same tumor specimen [38] displayed significant differences in stiffness, with HEY A8 cells being more compliant (Fig. 2 *a*). This finding is significant in that these isogenic cells also differ in their tumorigenicity in nude mice, whereby HEY A8 cells are more tumorigenic after intraperitoneal injection to nude mice [38]. To explore the relationship between mechanical properties of these ovarian cell lines and their metastatic potential, we examined the migratory and invasive properties of HEY and HEY A8 cells relative to IOSE using *in vitro* assays. HEY A8 cells displayed the greatest invasive and migratory activity followed by HEY cells and the IOSE control cells (Fig. 2 *b*) indicating that relative stiffness is inversely correlated with the indicators of metastatic potential (migration and invasiveness). These findings, summarized in Fig. 3 and Table 2, are consistent with previous reports linking cellular deformability with tumorgenic and metastatic potential [5], [7], [33].

**Table 2:** The strength of association between stiffness/migratory and stiffness/invasive properties expressed as Pearson’s product-moment (r) and Spearman’s rank (ρ) correlation coefficients and their p-values.

	Pearson		Spearman	
	r	p-val	ρ	p-val
**stiffness/** **relative** **migration**	−0.894	0.2956	−1	0.3333
**stiffness/** **relative invasion**	−0.9670	0.1641	−1	0.3333

### Gene Expression Profiling of HEY and HEY A8 Cells Links Increased Metastatic Potential with Changes in Actin-mediated Cytoskeletal Remodeling Pathways

Having established that the acquisition of decreased stiffness in HEY A8 cells relative to HEY cells is correlated with an increase in metastatic potential (i.e., cell migration and invasiveness), we performed a comparative gene expression analysis (DNA microarray) of these two cell lines in order to gain insight into the possible molecular basis of the acquired phenotype.

Using Significance Analysis of Microarrays (SAM) we identified 3,641 differentially expressed features between these cell lines (File F1) and determined that 1,258 genes were up-regulated and 1,272 down-regulated in HEY A8 relative to HEY cells (15 genes displayed discordant changes in expression between HEY and HEY A8 cells for redundant probe sets). Significantly enriched GeneGO pathways (Table 3) and process networks (Table 4) corresponding to our set of differentially expressed genes indicate that differences between HEY and HEY A8 cells include changes in mitotic phase of cell cycle (spindle assembly/chromosome separation, spindle microtubules), regulation of epithelial-to-mesenchymal transition (EMT), cytoskeletal remodeling, cell adhesion, and regulation of CFTR (cystic fibrosis transmembrane conductance regulator).

**Table 3:** Significantly enriched Genego Maps (p-val: p-value for hypergeometric distribution; ratio: number of mapped genes to total number of genes).

Map	p-val	Ratio
***Up- and down-regulated genes (FDR = 0.08)***		
Cell cycle: The metaphase checkpoint	1.548e-12	26/36
Cell cycle_Role of APC in cell cycle regulation	3.719e-11	23/32
Cell cycle_Chromosome condensation in prometaphase	2.895e-9	16/20
Cell cycle_Spindle assembly and chromosome separation	3.758e-9	21/32
Cell cycle_Role of Nek in cell cycle regulation	7.795e-6	16/29
Cell cycle_Start of DNA replication in early S phase	2.396e-5	16/31
Immune response_MIF - the neuroendocrine-macrophage connector	1.130e-4	15/31
Regulation of CFTR activity (norm and CF)	2.908e-4	17/40
Cell adhesion_ECM remodeling	3.202e-4	20/51
Reproduction_Progesterone-mediated oocyte maturation	7.001e-4	14/32
Development_Regulation of epithelial-to-mesenchymal transition (EMT)	1.070e-3	22/63
Cell adhesion_Plasmin signaling	1.443e-3	14/34
***Up-regulated genes (FDR = 0.07)***		
Cell cycle_The metaphase checkpoint	2.935e-19	26/36
Cell cycle_Role of APC in cell cycle regulation	4.422e-17	23/32
Cell cycle_Chromosome condensation in prometaphase	1.880e-13	16/20
Cell cycle_Spindle assembly and chromosome separation	3.278e-13	20/32
Cell cycle_Start of DNA replication in early S phase	4.183e-9	16/31
Cell cycle_Role of Nek in cell cycle regulation	1.204e-7	14/29
dCTP/dUTP metabolism	2.508e-4	13/45
Cell cycle_Initiation of mitosis	3.762e-4	9/25
Immune response_MIF - the neuroendocrine-macrophage connector	4.937e-4	10/31
Reproduction_Progesterone-mediated oocyte maturation	6.565e-4	10/32
Oxidative stress_Role of ASK1 under oxidative stress	7.419e-4	8/22
dATP/dITP metabolism	1.393e-3	13/53
wtCFTR and deltaF508 traffic/Membrane expression (norm and CF)	1.431e-3	8/24
Down-regulated genes (FDR = 0.14)		
Development_Regulation of epithelial-to-mesenchymal transition (EMT)	5.125e-5	16/63
Cell adhesion_Plasmin signaling	3.750e-4	10/34
Cytoskeleton remodeling_TGF, WNT and cytoskeletal remodeling	5.918e-4	20/107
Development_TGF-beta-dependent induction of EMT via RhoA, PI3K and ILK.	7.233e-4	11/43
Cell adhesion_ECM remodeling	9.434e-4	12/51
Development_PEDF signaling	1.233e-3	10/39

**Table 4:** Significantly enriched Genego Process Networks (p-val: p-value for hypergeometric distribution; ratio: number of mapped genes to total number of genes).

Network	p-val	Ratio
***Up and down-regulated genes (FDR = 0.04)***		
Cell cycle_Mitosis	5.493e-20	83/177
Cytoskeleton_Spindle microtubules	2.712e-16	56/108
Cell cycle_Core	6.026e-15	56/114
Cell cycle_G2-M	1.752e-10	73/204
Cell cycle_S phase	4.428e-8	53/147
Protein folding_Response to unfolded proteins	1.236e-5	27/68
Protein folding_ER and cytoplasm	6.199e-5	19/44
DNA damage_Checkpoint	9.910e-5	39/124
Inflammation_MIF signaling	2.442e-4	36/116
Cell cycle_G1-S	4.239e-4	46/163
Neurophysiological process_Circadian rhythm	4.631e-4	19/50
Reproduction_Progesterone signaling	5.458e-4	52/192
Muscle contraction_Nitric oxide signaling in the cardiovascular system	1.161e-3	27/86
Development_Regulation of angiogenesis	1.273e-3	54/208
Transcription_mRNA processing	1.652e-3	43/159
Cell cycle_Meiosis	2.033e-3	30/102
Protein folding_Folding in normal condition	2.288e-3	33/116
Cell adhesion_Integrin-mediated cell-matrix adhesion	2.454e-3	53/209
Development_EMT_Regulation of epithelial-to-mesenchymal transition	2.454e-3	53/209
DNA damage_BER-NER repair	3.018e-3	29/100
Cell cycle_G0–G1	3.849e-3	22/71
Cytoskeleton_Cytoplasmic microtubules	3.860e-3	32/115
Apoptosis_Apoptotic nucleus	5.767e-3	40/155
***Up-regulated genes (FDR = 0.04)***		
Cell cycle_Mitosis	1.026e-33	76/177
Cytoskeleton_Spindle microtubules	3.704e-26	52/108
Cell cycle_Core	7.111e-23	50/114
Cell cycle_G2-M	5.794e-19	63/204
Cell cycle_S phase	9.838e-16	48/147
Transcription_mRNA processing	8.204e-8	37/159
Cell cycle_G1-S	4.918e-7	36/163
DNA damage_Checkpoint	5.834e-7	30/124
DNA damage_BER-NER repair	2.709e-6	25/100
Cytoskeleton_Cytoplasmic microtubules	3.723e-5	25/115
Cell cycle_Meiosis	4.127e-5	23/102
Muscle contraction_Nitric oxide signaling in the cardiovascular system	2.527e-4	19/86
Protein folding_Folding in normal condition	3.250e-4	23/116
Apoptosis_Apoptotic nucleus	3.917e-4	28/155
DNA damage_MMR repair	4.318e-4	14/56
Cytoskeleton_Regulation of cytoskeleton rearrangement	1.094e-3	30/181
Protein folding_Response to unfolded proteins	1.128e-3	15/68
Protein folding_ER and cytoplasm	1.755e-3	11/44
DNA damage_DBS repair	1.893e-3	20/108
Protein folding_Protein folding nucleus	2.037e-3	13/58
Apoptosis_Apoptotic mitochondria	2.394e-3	15/73
Proteolysis_Ubiquitin-proteasomal proteolysis	2.524e-3	27/166
Reproduction_Progesterone signaling	5.399e-3	29/192
***Down-regulated genes (FDR = 0.07)***		
Development_EMT_Regulation of epithelial-to-mesenchymal transition	2.152e-5	36/209
Development_Regulation of angiogenesis	4.657e-5	35/208
Signal Transduction_TGF-beta, GDF and Activin signaling	1.730e-4	26/146
Cell adhesion_Platelet-endothelium-leucocyte interactions	2.006e-4	29/172
Inflammation_MIF signaling	5.717e-4	21/116
Neurophysiological process_Circadian rhythm	6.861e-4	12/50
Inflammation_Protein C signaling	7.018e-4	18/94
Blood coagulation	7.713e-4	17/87
Proliferation_Negative regulation of cell proliferation	1.615e-3	27/177
Reproduction_FSH-beta signaling pathway	3.870e-3	23/152
Proteolysis_ECM remodeling	4.364e-3	15/85
Proteolysis_Connective tissue degradation	4.593e-3	19/119
Signal transduction_Androgen receptor signaling cross-talk	4.827e-3	12/62
Translation_Elongation-Termination	5.344e-3	22/147

**Figure 4:**
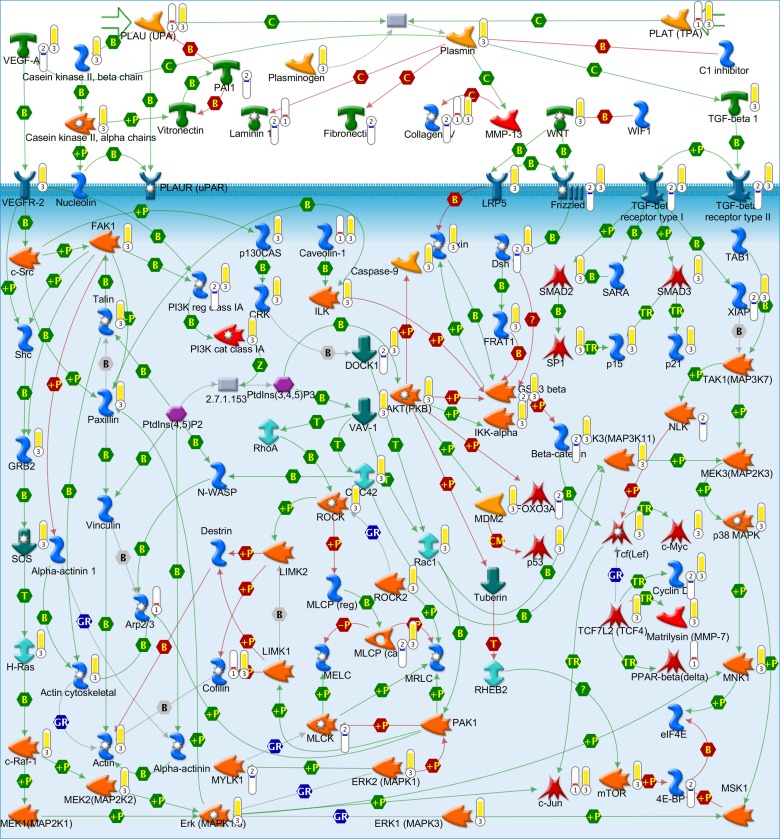
TGF, WNT and cytoskeletal remodeling GeneGO pathway. Red thermometer: genes transcriptionally up-regulated in HEY A8 cells; blue thermometer: genes transcriptionally down-regulated in HEY A8 cells; yellow thermometer: proteins topologically relevant to the set of up-regulated genes.

**Figure 5:**
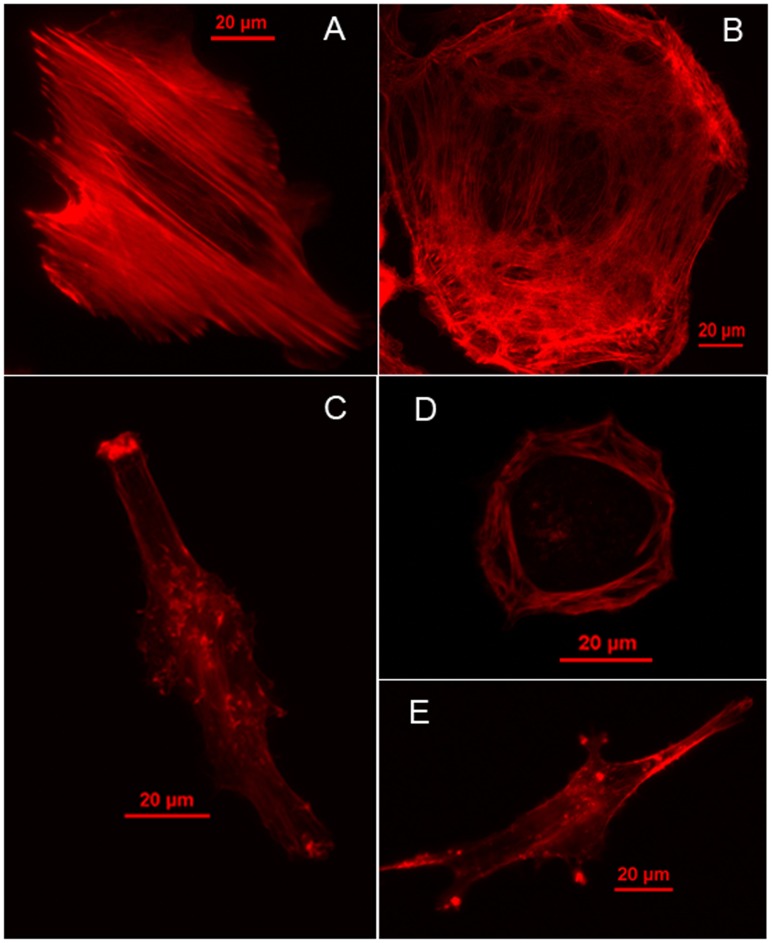
Fluorescence images of F-actin. (A) IOSE, (B) OVCAR-4, (C) HEY, (D) OVCAR-3 and (E) HEY A8.

**Figure 6:**
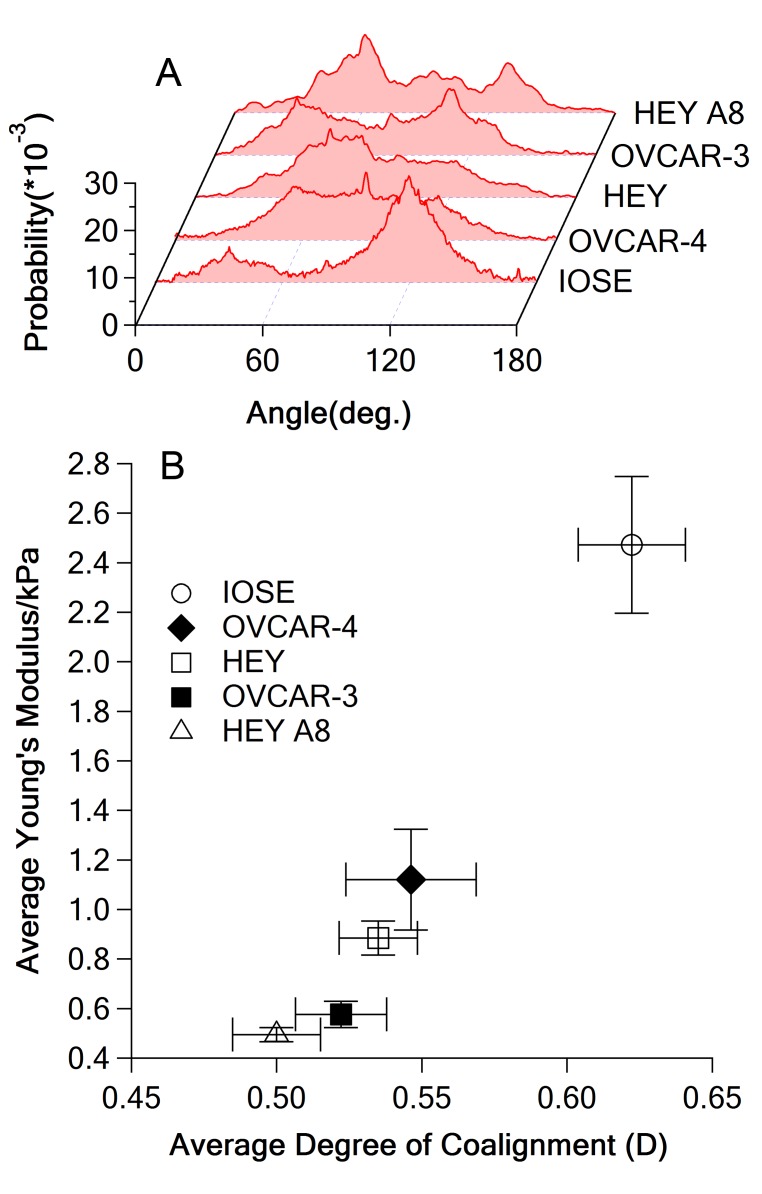
Relationship of F-actin coalignment and stiffness. (A) Representative orientation distribution function for each cell line, (B) stiffness versus degree of coalignment of F-actin, error bar represents standard error of mean (SEM).

To further explore the potential biological significance of the differences in gene expression between HEY and HEY A8 cells, we employed topological significance analysis (TSA). Using this approach we identified 1,108 unique Entrez Gene IDs corresponding to topologically relevant proteins associated with up-regulated genes in HEY A8 cells (Entrez gene IDs converted to the 1,199 official gene symbols are presented in File S2). Significantly enriched GeneGO pathways for these topologically relevant proteins (360 pathways at FDR = 0.26%) suggest considerable biological differences between HEY and HEY A8 cells including those that could not be identified on transcriptional level (File S3). A full discussion of these results is beyond the scope of this paper and will be presented elsewhere. Here we will limit our discussion to those changes most relevant to the observed differences in cell stiffness discussed above.

The top scoring GeneGO pathway for topologically relevant proteins (File S3) is the “TGF, WNT and cytoskeletal remodeling pathway” (Fig. 4). This pathway was also identified as significantly enriched for genes down-regulated in HEY A8 cells (Table 3). These findings indicate that differences in stiffness between HEY and HEY A8 cells are related to cytoskeletal remodeling. Our findings support earlier predictions that the basis of reduced stiffness associated with cancer [39] and highly invasive cells [40] may be associated with cytoskeletal remodeling. Further support for this conclusion comes from TSA which found various isophorms of actin superfamily topologically significant for genes up-regulated in HEY A8 cells (File S2), as well as from differential expression analysis, which found that the actin-monomer-binding proteins CFL2, TWF1 and PFN1 that regulate the incorporation of actin monomers into filaments [41] are overexpressed in HEY A8 cells. Overexpression of cofilin-2 (CFL2) enhances the rate of actin filament turnover by depolymerizing filaments at their pointed ends [42] while twinfilin (TWF1) functions as an actin-monomer-sequestering protein that also severs actin filaments to promote filament disassembly *in vitro* and its rapid turnover *in vivo* [43]. The fact that myosin light chain kinase (MYLK), which promotes an actin-activated myosin motor activity and tension generation [44], is significantly down-regulated in HEY A8 cells further supports the role of stress fibers in observed difference in cell stiffness. Interestingly, BDM and ML-7, the inhibitors of MYLK, have been previously reported to induce softening of fibroblast cell lines [45]. Phosphorylation of myosin light chain, stress fiber formation, and subsequent increase in cell stiffness can be also induced via RhoA/Rho kinase pathway [46], which is inactivated by cAMP-dependent protein kinase (PKA) [47]. We found that regulatory (PRKAR2A, PRKAR2B) and catalytic (PRKACB) subunit genes of PKA are significantly overexpressed in HEY A8 relative to HEY cells suggesting PKA-dependent inactivation of RhoA/Rho kinase pathway in HEY A8 cells. qPCR validation of differential gene expression data from microarray experiment confirmed overexpression of PRKAA2 and TWF1 genes with respective fold changes 3.89 and 2.63 and decreased expression of MYLK gene with fold change −2.0 in HEY A8 cells relative to HEY cells (Fig. S2).

### Microscopic Analyses of Ovarian Cancer and Control Cells Confirm that Actin-mediated Cytoskeletal Remodeling is Associated with Change in Metastatic Potential

Since our molecular analyses indicated that actin-mediated cytoskeletal remodeling may be a major contributor to the observed differences in cell stiffness between HEY and the more invasive/tumorigenic HEY A8 cells, we tested the hypothesis by examining the cytoskeletal structure of HEY and HEY A8 cells relative to other ovarian cancer cells (OVCAR-3, OVCAR-4) and non-malignant immortalized ovarian surface epithelial cells (IOSE). The results presented in Fig. 5 display denser, well-aligned F-actin with longer stress fibers in IOSE relative to all of the ovarian cancer cells. This is consistent with previously reported comparisons between normal and cancer cells [5], [20].

In addition, the degree of co-alignment of F-actin fibers among the various examined cell lines correlated with the observed difference in cells stiffness. For example, for the stiffer IOSE cells, the actin filaments are distributed through the cell body, with most F-actin bundles aligned along the long axis of the cell with well-defined stress fibers and focal contacts. In contrast, actin filaments in the softer ovarian cancer cells are less organized and F-actin bundles are oriented randomly with disrupted, short segments. In OVCAR-4 cells, actin filaments are aligned only locally and form a tangled network. In HEY cells, the actin filaments break into shorter segments and display reduced co-alignment. F-actin in OVCAR-3 cells maintains a cortical structure with most filaments lying in the peripheral region of the cell, though at a relatively low density. HEY A8 cells show similar characteristics of F-actin distribution to HEY cells, but with a lower density. Since the actin cytoskeleton contributes to the mechanical properties of the cells, observed variations are consistent with differences in cell stiffness and with previous reports [8], [19].

We quantitatively analyzed the degree of co-alignment of F-actin in all five cell lines by using orientation distribution function. The results are displayed in Fig. 6 *a*. A parameter *D*, defined as 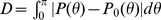 was used to quantify the degree of co-alignment of F-actin from the orientation distribution functions, where *P(θ)* represents the orientation distribution function, which is the probability for a fiber oriented at the angle of *θ* in the fluorescence image. *P_0_(θ)* is the orientation distribution function for the extreme case where F-actin are completely randomly distributed and therefore has a value of *P_0_(θ) = *1/180. In the extreme case in which all actin fibers are oriented randomly without any preference, the orientation distribution function should be a constant and independent of angle. From the definition of *D*, a higher value of *D* indicates an increasing deviation from random alignment and a higher degree of co-alignment of F-actin. Orientation distributions of F-actin differ among the five ovarian cell lines (Fig. 6 *a*). In non-malignant IOSE cells, most of F-actin bundles were aligned along the long axis of the cell, which is ∼135°. In contrast, OVCAR-4 cells display several orientations of F-actin, indicating that actin filaments are neither uniformly aligned, nor evenly distributed. This result is readily apparent from the fluorescence image in Fig. 5. The relationship between the degree of co-alignment of F-actin and stiffness of single cell is plotted in Fig. 6 *b*, which shows strong and significant positive correlation (r = 0.99834 with p-val = 8.064×10^−5^ and ρ = 1 with p-val = 0.01667). This result suggests that changes in co-alignment of F-actin bundles could also contribute to the differences in cell stiffness within the examined ovarian cell lines.

### Conclusions

Our analysis of non-malignant IOSE and four ovarian cancer cell lines indicates that cancer cells exhibit a lower mean stiffness relative to non-malignant precursor cells. Interestingly, we also find that the increase in invasive and migratory capacity associated with HEY A8 cells relative to HEY cells is also correlated with a significant reduction in cell stiffness. Comparative gene expression analysis of HEY A8 and HEY cells indicates that the molecular basis of the reduction in stiffness between these cells is reflective of extensive molecular changes including changes in actin cytoskeleton remodeling pathways. Microscopic analyses of actin cytoskeleton in ovarian cancer and control cells are consistent with this hypothesis.

Since our measurements are conducted with cancer cell lines, further studies will be needed to see if similar results are found in the case of patient-derived cells. Establishing the relative metastatic potential of cancer cells is an important factor in the design of optimal strategies in the personalized treatment of cancer [48]. Currently, extensive molecular profiling is required to estimate the metastatic potential of cancer cells [49]. Collectively, our results indicate that mechanical stiffness may be a useful biomarker in the development of accurate, non-invasive clinical methods to evaluate the relative metastatic potential of ovarian and perhaps other types of cancer cells. Stiffness may be particularly important as a biomarker with the development of rapid biomechanical assaying techniques [50], [51].

## Supporting Information

Figure S1 **Calculation of the orientation distribution function from fluorescence image and Fast Fourier Transform.** (A) Original fluorescence image, (B) mesh representation of the transformed image, and (C) orientation distribution function.
(TIF)

Figure S2 **Results of qPCR validation of microarray gene expression data for selected genes.** FC: gene expression fold changes in HEYA8 relative to HEY cells. Error bars: standard errors of means, N = 3. *: p<0.05; **: p<0.01 (Student’s t-test).
(TIF)

File S1 Results of differential expression analysis by Significance Analysis of Microarrays (SAM). Using Significance Analysis of Microarrays (SAM), 3,641 differentially expressed features were identified between HEY A8 and HEY cell lines (FDR = 1.1% and |FC|≥1.5). 1,258 genes were up-regulated and 1,272 genes were down-regulated.
(XLS)

File S2 List of proteins topologically relevant to genes up-regulated in HEY A8 cells. Topologically relevant proteins (represented by 1199 official gene symbols) were identified by topological significance analysis (p<0.01).
(XLS)

File S3 GeneGO pathways and significance. 360 significantly enriched GeneGO pathways (FDR = 0.26%) for topologically relevant proteins suggest considerable biological differences between HEY and HEY A8 cells.
(XLS)
