# Supplementary figures and images for: Cell Stiffness Is a Biomarker of the Metastatic Potential of Ovarian Cancer Cells
Source: PLoS One. 2012 Oct 4;7(10):e46609. doi: 10.1371/journal.pone.0046609 (PMC3464294; doi:10.1371/journal.pone.0046609)

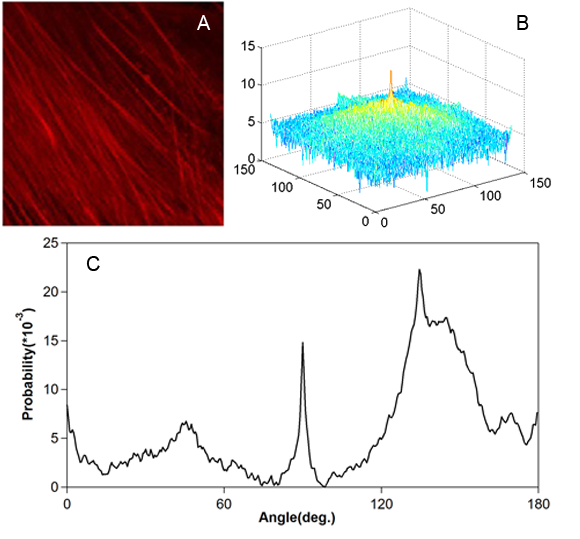

Supplement: Figure S1 — Calculation of the orientation distribution function from fluorescence image and Fast Fourier Transform. (A) Original fluorescence image, (B) mesh representation of the transformed image, and (C) orientation distribution function. (TIF) [file pone.0046609.s001.tif]

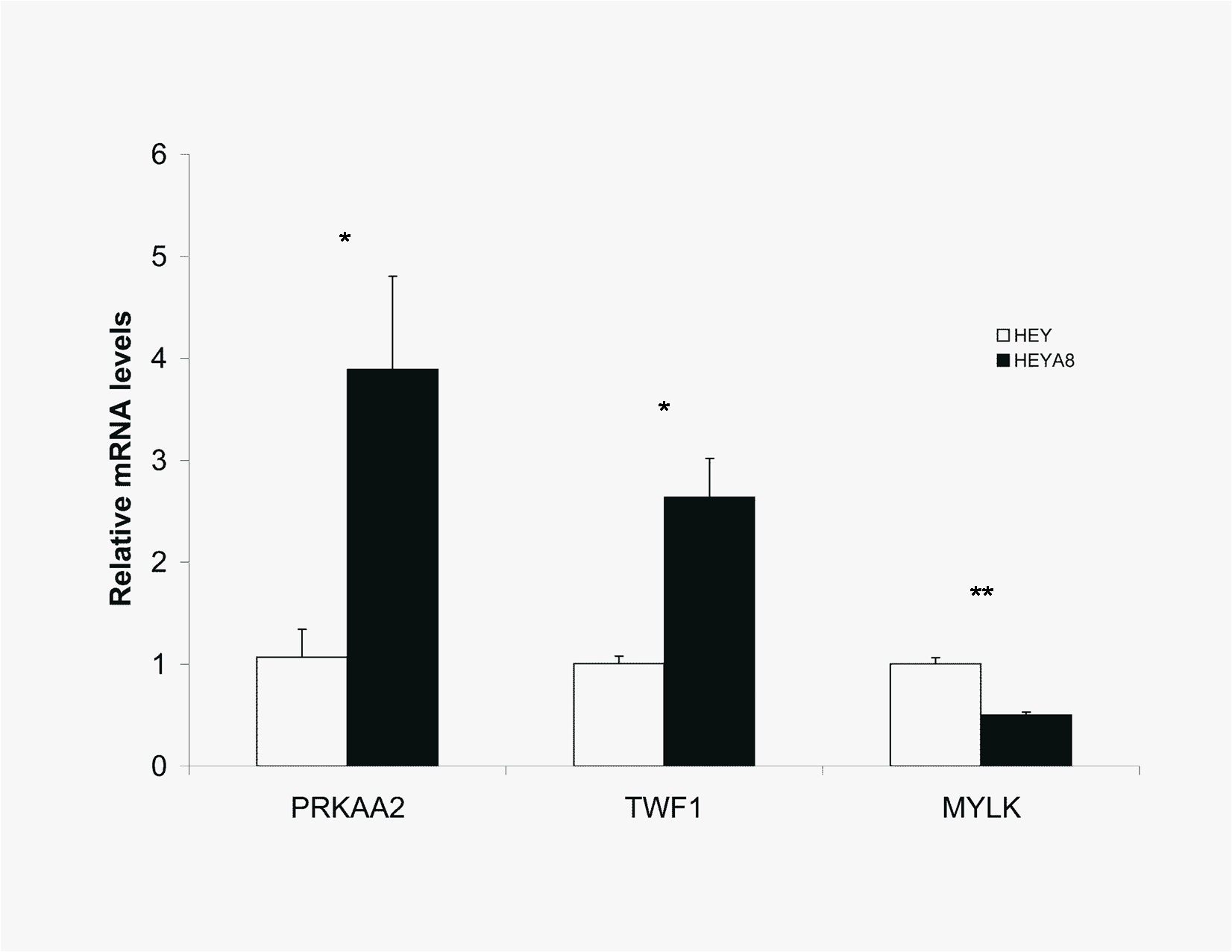

Supplement: Figure S2 — Results of qPCR validation of microarray gene expression data for selected genes. FC: gene expression fold changes in HEYA8 relative to HEY cells. Error bars: standard errors of means, N = 3. *: p<0.05; **: p<0.01 (Student’s t-test). (TIF) [file pone.0046609.s002.tif]
